# Thrombasthénie de Glanzmann et grossesse: à propos d'un cas et revue de la littérature

**Published:** 2011-12-21

**Authors:** Myriam Rachad, Hikmat Chaara, Fatim Zahra Fdili, Sofia Jayi, Hakimat Bouguern, Moulay Abdelilah Melhouf

**Affiliations:** 1 Service de gynécologie-obstétrique II, CHU Hassan II, Fès, Maroc

**Keywords:** Thrombasthénie de Glanzmann, grossesse, complications, Maroc

## Abstract

La thrombasthenie de Glanzmann est une anomalie plaquettaire qualitative, à transmission autosomale récessive, due à un déficit en glycoprotéine IIb/IIIa (GPIIb/IIIa), avec un défaut d'agrégation plaquettaire. Il en résulte une perturbation de l'hémostase primaire, à l'origine d'hémorragie plus ou mois importantes. Nous rapportons l'observation de N.A, âgée de 32 ans, suivie depuis l'enfance pour maladie de Glanzmann, troisième pare, avec deux accouchements par voie basse et une césarienne programmée, compliqués tous les trois par hémorragie de délivrance, jugulée avec succés. La grossesse chez une patiente thrombo-asthenique reste rare, peu de cas jusqu'à présent, sont décrits. A travers cette observation, et à la lumière d'une revue de la littérature, nous allons essayer surtout, de mettre le point sur la prise en charge de cette association délicate, qui met en jeu, à chaque fois, le pronostic vital maternel.

## Introduction

La Thrombasthénie de Glanzmann est une maladie congénitale rare, dont la prévalence n'est pas bien connue. Elle est due à une anomalie qualitative ou quantitative du complexe glycoproteique : GPIIb/IIIA avec comme conséquence un défaut d'agrégation plaquettaire [1]. La grossesse chez une patiente thrombo-asthénique, est souvent émaillée d'hémorragies d'origines diverses, surtout en per et post partum immédiat, périodes particulièrement critiques. L'objectif de ce travail est de montrer la gravité et la difficulté de la gestion de la thrombasthénie de Glanzmann au cours de la grossesse, en insistant essentiellement sur le mode d'accouchement et l'actualité thérapeutique de prise en charge de l'hémorragie.

## Observation

Mme NA, 32 ans, née de parents consanguins, connue comme ses deux sœurs porteuses de la thrombasthenie dès le bas âge. Ses antécédents étaient marqués à l'enfance par des ecchymoses, un syndrome hémorragique (épistaxis, gingivorragie), nécessitant des transfusions répétées, et à l’âge pubertaire par les ménorragies jugulées par un traitement progestatif de synthèse. Le dosage des anticorps anti GPIIb-IIIa n'a pu être réalisé par manque de moyens. Mariée depuis 10 ans, sans notion de consanguinité. C'est une troisième geste. La première grossesse, comme la deuxième, étaient non suivies, marquées par la survenu de quelques épisodes de gingivorragies et d’épistaxis. Admise chaque fois, en pleine phase active du travail, l'accouchement par voie basse a été accepté. Compliqué par la survenu d'hémorragie de délivrance, jugulée par des moyens mécaniques (révision utérine, tamponnement, sac de sable) et médicaux (uterotoniques). La patiente n'a pas nécessité de transfusion, ni au cours de la grossesse, ni en per ou post partum. Les nouveaux nés étaient tous les deux de sexe masculin pesant respectivement 3300g pour le premier et 3900g pour le deuxième. Les NFS initiales réalisées à J1 de vie étaient normales et l'examen clinique était sans particularité. Actuellement, ils sont âgés de 9 et 5 ans, indemnes de la maladie. Lors de sa troisième grossesse, la patiente ne s'est présentée à la consultation prénatale qu′à 36 SA. Elle ne rapporte pas de syndrome hémorragique durant cette grossesse. L'examen clinique était sans particularité, l’échographie obstétricale objective: toutes les mensurations au 50éme percentile de 36 SA avec un liquide amniotique normal. La numération formule sanguine était normale. Une hospitalisation pour mise au point a été décidée à 38SA, et après discussion multidisciplinaire: obstétriciens, réanimateurs et hématologues, une césarienne programmée a été indiqué à 39 SA. Au cours de l'acte, la patiente a présenté une hémorragie de la délivrance, jugulée par 60 UI de synthocynon, 5 cp de mesoprostol 200 mg en intrarectal, ligature vasculaire. Vue l'hémorragie importante mettant en jeu le pronostic maternel, elle a été transfusée par 2 culots globulaires et 6 culots plaquettaires standards, dans l'indisponibilité de culots HLA compatibles. Une ligature section tubaire a été faite. La patiente a été transférée, par la suite, dans un service de réanimation avec évolution favorable.

Le nouveau né était de sexe féminin de 3700 grammes, avait une thrombopénie à 118000 éléments /mm, sans syndrome hémorragique. Hospitalisé 5 jours en néonatologie pour surveillance. Son taux de plaquettes était normal à la sortie.

## Discussion

La thrombasthénie de Glanzmann est une entité rare, décrite la première fois en 1918 par un pédiatre Suisse, [2] dont elle porte le nom. Son incidence reste actuellement encore inconnue. Cette maladie est caractérisée par une absence d'agrégation des plaquettes quel que soit l'agoniste utilisé [3]. Elle est liée à un déficit quantitatif en site d'amarrage du fibrinogène: les complexes GPIIB/IIIA ou intégrines 45; IIb 46; 3 dont il existe 40000 à 80000 copies par plaquette normale [4]. Ce chiffre est réduit à moins de 50% dans les variantes dits de type III, à moins de 20% dans le type II, forme atténuée et à moins de 5% dans le type I, forme sévère, la plus fréquente. [4] Il existe des variantes ou l'anomalie du complexe est strictement qualitative. Les gènes codant pour 45; IIb 46;3 sont situés sur le chromosome 17 q21-23 [4]. La biologie moléculaire a permis l’étude des anomalies génétiques associées. La première anomalie a été décrite en 1990 et depuis près de 20 anomalies en été définies (mutations ponctuelles, délétions, inversion, etc.) [5], donnant naissance à des nouvelles classifications. [6].Cependant aucune de ces classifications ne permet de prédire fidèlement la gravité du syndrome hémorragique [7].

Il s'agit d'une maladie héréditaire, transmise sur un mode autosomal récessif et qui semble plus fréquente dans les ethnies à forte consanguinité comme les indiens, iraniens, palestiniens et jordaniens arabes, voire les populations d'origine rom [1]. La plupart de temps, la maladie est diagnostiquée dans la petite enfance avant l’âge de 5 ans, mais peut être aussi à un âge avancé [8]. Ses manifestations cliniques sont hémorragiques, cutanéomuqueuses à type d’épistaxis, gingivorragies, hématuries, pétéchies, ménométrorragies et hémarthroses [1,8]. Le diagnostic biologique est fait sur un temps de saignements (TS) allongé supérieur à 20 minutes, une agrégation plaquettaire nulle quel que soit l'inducteur physiologique utilisé (ADP, collagène, thrombine, acide arachidonique, épinephrine), l'absence de thrombopénie et d'anomalie morphologique des plaquettes [9]. Le diagnostic différentiel doit être fait avec les autres anomalies plaquettaires, essentiellement, la maladie de Bernard soulier et le pseudo Willebrand [9]. L'association Glanzmann et grossesse est une association assez rare, qui pose toujours le problème de prise en charge du risque hémorragique que ce soit au cours de la grossesse, ou au cours de l'accouchement et du post partum immédiat, périodes les plus critiques. Aucune étude jusqu′à présent ne contre-indique la grossesse chez les femmes porteuses de cette thrombasthénie. De 1963 à 2008, les publications font état de 96 accouchements chez des femmes thrombo-asthéniques, dont seules 38 observations étaient détaillées [10–13]. Au cours de la grossesse, il n'existe pas de risque hémorragique majeur, quelques cas de gingivorragies et d’épistaxis ont été rapportés, jugulés par un traitement local (compression, tamponnement) ou par anti fibrinolytique (acide tranéxamique, acide aminocarpoique). Néanmoins, il semble licite de compenser au mieux la carence martiale. Dans une seule observation, la patiente a nécessité des transfusions répétées par des culots globulaires et des culots plaquettaires à partir de la 28^e^ semaine d'aménorrhée, à cause des gingivorragies importantes [14]. Il n'existe pas de consensus sur le mode d'accouchement, dans la littérature: 11 patientes ont accouché par voie basse, 12 par césarienne (dans les autres cas le mode d'accouchement n’était pas précisé) [3,10–13]. Pour certains auteurs, l'accouchement par voie basse est moins hémorragique, la césarienne ne sera donc pratiquée qu'en cas d'indication obstétricale. Pour d'autres la césarienne est plus sûre, car elle permet de programmer précisément l'accouchement afin d'appliquer de façon optimale la stratégie multidisciplinaire de prise en charge, impliquant: obstétricien, réanimateur et hématologue. La fréquence des hémorragies en per et post partum est la même quelle que soit la voie d'accouchement. Ce qui est bien illustré par notre observation, ou la patiente a présenté l'hémorragie de délivrance, après accouchements par voie basse mais également et de manière plus grave au cours de la césarienne programmée. Pendant plusieurs années, le traitement de première intention des hémorragies; y compris l'hémorragie de la délivrance, résistant aux traitements locaux et aux antifibrinolytiques; a été la transfusion par les culots plaquettaires [3], avec le risque de développer des anticorps (anti GP IIb-IIIa ou HLA), dont la prévalence reste inconnue. Lorsque ces anticorps existent, la transfusion des plaquettes, par la suite, peut devenir rapidement inefficace. Il faut alors avoir recours à des échanges plasmatiques ou idéalement à des séances d'immunoabsorption [15]. Au cours de la dernière décennie, l'intérêt des traitements hémostatiques, notamment du facteur VII activé recombinant, dans la lutte contre l'hémorragie de la délivrance chez les patientes thrombasthéniques a été souligné dans la littérature [4]. Utilisé hors AMM, il a été proposé avec succès dans les cas d’échec ou d'inefficacité des transfusions prophylactiques. Son efficacité reste à évaluer, du fait de la diversité des protocoles utilisés et du nombre limité de patient inclus dans un registre (n=30) [4]. D'autres thérapeutiques ont été également proposées mais avec une efficacité limitée à savoir: la corticothérapie [12], qui reste à faible effet sur les AC anti HLA. Ces derniers baissent spontanément à distance des transfusions de culots plaquettaires; les prostaglandines et les ocytocique [16]; l'estrogènothérapie en post-partum tardif, pour prévenir les métrorragies [16].

Dans notre observation, on a accepté l'accouchement par voie basse, les deux premières grossesses car la patientes a été admise aux urgences en pleine phase active de travail. L'hémorragie de la délivrance a pu être heureusement jugulée par les utérotoniques et les moyens physiques (tamponnement). Pour la troisième grossesse et pour d'optimiser la prise en charge, une césarienne programmée a été indiquée, cependant elle a été compliquée par une hémorragie traitée, dans la limite de nos moyens: ocytociques, misoprostol intrarectal, ligature vasculaire et après échec, transfusion par des culots plaquettaires, et globulaires.

Le retentissement fætal de cette thrombasthénie semble relativement peu important [17]. Chez le nouveau-né, par définition hétérozygote (lorsque le couple n'est pas consanguin et que le père ne présente pas de pathologie plaquettaire), le risque hémorragique est faible voire nul. Cependant à cause du passage transplacentaire des anticorps anti GPIIbIIIa chez les mères immunisées, le nouveau-né peut développer une thrombopénie, souvent transitoire ne nécessitant pas de traitement particulier. Elle peut entrainer, dans des cas rares, une hémorragie intracérébrale [11]. La numération plaquettaire est impérative à la naissance et dans les semaines qui suivent [17]. Dans notre observation, la thrombopénie néonatale, transitoire, n'a intéressé que le troisième nouveau-né, témoignant alors indirectement d'une immunisation plaquettaire de la mère.

## Conclusion

La prise en charge de la grossesse chez la femme thrombasthénique reste toujours délicate. Le pronostic vital peut être engagé surtout en péripartum. Le mode d'accouchement est très discuté. En cas d'hémorragie, les traitements locaux doivent être privilégiés. Les transfusions par culots plaquettaires sont déconseillées, en dehors de l'extrême urgence, afin d’éviter l'apparition des anticorps anti plaquettaires. Le facteur VII pourrait être une alternative intéressante. Chez le nouveau-né, le risque majeur reste la thrombopénie, souvent transitoire, peut être parfois responsable d'hémorragie sévère. Il est donc recommandable de prendre en charge ces patientes dans un centre multidisciplinaire, capable la fois de gérer les complications maternelles, mais aussi néonatale de cette association qui fait toujours peur.
